# The C-terminal α-helix is crucial for the activity of the bacterial ABC transporter BmrA

**DOI:** 10.1016/j.jbc.2024.108098

**Published:** 2024-12-18

**Authors:** Veronika Osten, Kristin Oepen, Dirk Schneider

**Affiliations:** 1 Department of Chemistry – Biochemistry, Johannes Gutenberg-University, Mainz, Germany; 2 Institute of Molecular Physiology, Johannes Gutenberg-University, Mainz, Germany

**Keywords:** ABC transporter, multidrug transporter, membrane transport, ATPase, mutagenesis, dimerization, P-glycoprotein

## Abstract

ABC transporters are membrane integral proteins that consist of a transmembrane domain and nucleotide-binding domain (NBD). Two monomers (half-transporters) of the *Bacillus subtilis* ABC transporter *Bacillus* multidrug-resistance ATP (BmrA) dimerize to build a functional full-transporter. As all ABC exporters, BmrA uses the free energy of ATP hydrolysis to transport substrate molecules across the cell membrane. For substrate transport, a BmrA dimer undergoes major conformational changes. ATP binding drives dimerization of the NBDs followed by the hydrolysis of the nucleotides. Conserved structural elements within the NBD and transmembrane domain are crucial for dimerization and the activity of BmrA. In the BmrA structure, an α-helix is present at the C-terminus, which can be subdivided in two smaller helices. As shown here, the very C-terminal helix (fragment) is not crucial for the BmrA activity. In fact, based on Cys-scanning mutagenesis, this region is highly flexible. In contrast, a BmrA variant lacking the entire C-terminal α-helix, showed no ATPase and transport activity. Via Ala-scanning, we identified residues in the N-terminal fragment of the helix that are crucial for the BmrA activity, most likely *via* establishing contacts to structural elements involved in ATP recognition, binding, and/or hydrolysis.

ABC transporters are found in all domains of life (1, 2). These transporters either import (mainly in bacteria and plants) or export a broad variety of substrates (3, 4). Structurally, ABC transporters consist of four core domains: two transmembrane domains (TMDs) and two cytosolic nucleotide-binding domains (NBDs), albeit the exact molecular architecture of these transporters is extremely diverse (3, 5, 6). In fact, a transporter can consist of a single polypeptide chain that contains all four domains, or, if one TMD and one NBD are located on a single polypeptide chain, two different of such half-transporters heterodimerize, such as the ABC transporter TAP1/TAP2 (7), or two identical half-transporters homodimerize (*e.g.*, Sav1866, (8)) to form an active transporter. Finally, each of the four domains can be individual proteins, which assemble to form a functional ABC exporter (5, 9).

The structurally diverse substrates of ABC exporters enter the substrate-binding cavity, which is located in the TMD (3, 10), either from the cytoplasmic site of the membrane or from within the membrane (11, 12). Upon substrate binding, the transporter undergoes major conformational changes, finally resulting in transmembrane transport of a substrate against a concentration gradient (5, 8, 13, 14, 15). Transmembrane substrate transport is fueled by ATP hydrolysis at the NBDs, which are located at the cytoplasmic side. The sequences and topologies of NBDs of different ABC transporters are highly similar and motifs involved in ATP recognition, binding, and hydrolysis are conserved (6, 16, 17). These motifs within the NBDs are located in two different subdomains of the NBD, an α-helical domain with the ABC signature motif and the X-loop, and a RecA-like subdomain with the Walker A and Walker B motifs, Q-, D-, and H-loop. The ATP-binding sites are formed *via* interaction of two NBDs, and an ATP molecule is bound between the Walker A motif of one NBD and the ABC signature motif of the opposing NBD (18, 19). Consequently, ATP binding triggers dimerization of the two opposing NBDs of an ABC transporter (20, 21, 22, 23, 24, 25). The coupling helices at the cytoplasmic side of the protein are additionally relevant for NBD-TMD communication (8, 26, 27). ATP-induced NBD dimerization causes a conformational change of the exporter from an inward-facing conformation (IF) *via* an occluded state to an outward-facing conformation (OF) (28). Now, the TMDs cavity is open to the extracellular side of the membrane and the substrate is released. Substrate release and ATP hydrolysis set the transporter back into the IF conformation and the transporter is ready for the next translocation cycle (29).

Isolated NBDs have typically no propensity to dimerize in the absence or presence of ATP (30), and, in fact, the TMDs facilitate NBD dimerization upon ATP binding. Occasionally, NBDs of transporters contain additional domains that stabilize the dimer also in the absence of ATP, as for example, the C-terminal regulatory domain (of 136 residues) of the ABC importer MalK (31, 32). Furthermore, the NBDs of the tetrameric ABC transporter ModB_2_C_2_ are suggested to be in constant contact, mediated by α-helices at the C-terminal ends that interact to some degree and thereby link the NBDs (33). Likewise, the homodimeric ABC transporters Atm1 (34) and HlyB (35) appear to have interacting helices at their NBDs C-termini, which were postulated to stabilize the dimer. Since many ABC exporters appear to have such short C-terminal α-helices, this structure might more generally contribute to dimer stability (36). However, the exact role of these short C-terminal helices in the translocation cycle still is enigmatic, although the C-terminal helices appear to be important for the activity of the heterodimeric ABC exporter TmrAB (37). Also the homodimeric ABC transporter *Bacillus* multidrug resistance ATP (BmrA) of the Gram-positive bacterium *Bacillus subtilis* contains an α-helical segment at its very C-terminus (38, 39). BmrA can extrude a broad variety of substrates out of a membrane, and the protein shows sequence identity with the human P-glycoprotein as well as with the bacterial ABC exporters MsbA and LmrA (40). Not only because of this, BmrA became a paradigm for studying ABC transporter structure, function, and multidrug resistance. The structure of the BmrA exporter in the OF conformation was solved *via* X-ray crystallography as well as cryo-EM (41). As can be seen in these structures (Fig. 1, *A* and *B*), the α-helical C-terminal end of the NBDs are in close distance, and thus, potentially interact. Recently, the IF conformation of wt BmrA was also solved *via* cryo-EM and here the C-terminal ends remained in close distance (42).

**Figure 1:**
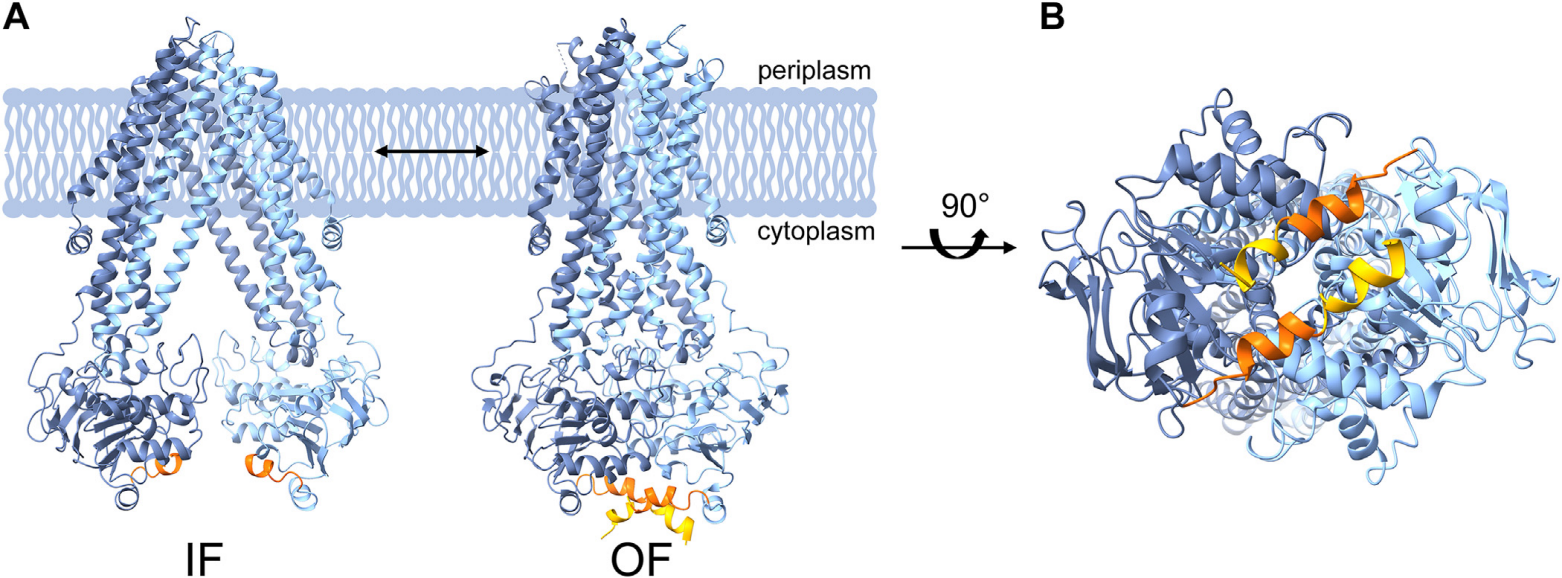
**The ABC transporter BmrA in the inward-facing and outward-facing conformation**. *A*, a BmrA dimer (half-transporters: *light blue and dark blue*) in a lipid bilayer with the first C-terminal α-helical segment of the NBDs highlighted in *orange* and the second segment highlighted in *yellow*. The inward-facing (IF, pdb: 8QOE) and the outward-facing (OF, pdb: 6R81) conformations are shown. *B*, view on the C-termini from the cytoplasmic side after a 90° rotation of the BmrA dimer in its OF conformation as shown in (*A*). BmrA, *Bacillus* multidrug resistance ATP.

In the present study, we show that deletion of the entire C-terminal α-helices of BmrA leads to a complete loss of the ABC transporter activity, measured as ATPase activity as well as *via* quantifying transport of the dye Hoechst 33342 across membranes. However, the C-terminal helix is not continuous, yet in fact is built from two smaller α-helical segments. Deletion of the most C-terminal α-helical fragment did not affect the BmrA activity, whereas the N-terminal helix part is crucial for the BmrA ATPase as well as transport activity. In fact, *via* Cys-crosslinking, we show that the C-terminal part is rather flexible. In contrast, upon replacing individual residues in the N-terminal fragment of the α-helix, we identified residues which are crucial for the BmrA activity, most likely due to interactions with structural elements crucial for ATP binding and/or hydrolysis.

## Results

### The BmrA C-terminus is crucial for activity

In the BmrA structure, a short α-helix is located at the very C-terminus (41), and the two C-terminal helices of two interacting monomers (half-transporters) appear to interact in an anti-parallel fashion in the dimer (Fig. 1). Based on the previous observations that many ABC exporters contain short α-helices at the C-terminal end, which might contribute to the dimer stability (31, 32, 33, 34, 35, 36), it is reasonable to assume that the C-terminal α-helix might also be crucial for the activity of BmrA. Therefore, we analyzed in the present study (i) whether the BmrA C-terminal helices are crucial for the BmrA activity and (ii) whether the C-termini of adjacent BmrA half-transporters indeed interact.

**Figure 2:**
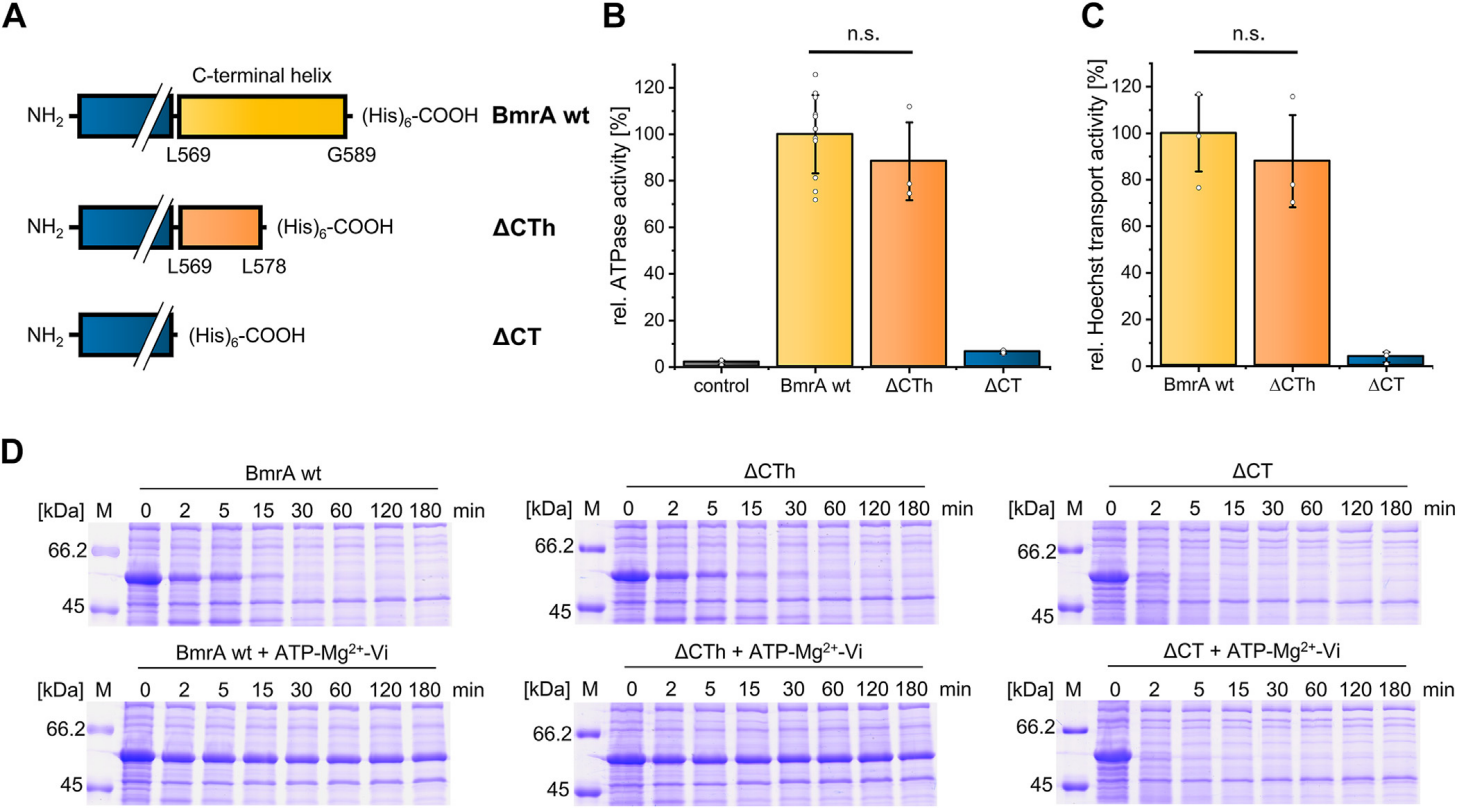
**ATPase, Hoechst 33342 transport activities, and limited proteolysis of C terminally truncated BmrA variants**. *A*, sketch of the C-terminal end showing the ΔCTh and the ΔCT compared to BmrA wt. *B*, mean of relative ATPase activity of BmrA wt and variants (n = 10 for the wt, n = 3 for the truncated variants, ± SD, two-sample *t*-test, *p* = 0.61). Individual data points are shown and represent independent biological replicates. *C*, mean of relative Hoechst 33342 transport activity assay of IMVs containing overexpressed BmrA wt or variants (n = 3, ± SD, two-sample *t* test, *p* = 0.50). Individual data points are shown and represent independent biological replicates. *D*, SDS-PAGE analysis of IMVs containing overexpressed BmrA wt, ΔCTh, or ΔCT after limited digestion by trypsin. Samples in presence or absence of ATP, Mg^2+^, and orthovanadate, which stabilizes the OF conformation, are shown. Samples were treated for the indicated times (0, 2, 5, 15, 30, 60, 120, and 180 min) with trypsin and subsequently analyzed *via* SDS-PAGE. BmrA, *Bacillus* multidrug resistance ATP; IMV, inverted membrane vesicle; OF, outward facing.

To investigate the putative role of the C-terminal helix on the BmrA activity, we first analyzed BmrA variants where the C-terminal α-helix was completely absent (residues L569-G589, ΔCT) or solely the very last α-helical region (residues K579-G589, ΔCTh, ”h” for half) that forms an independent helix in some structures (Figs. 1, *A* and *B* and 2*A*).

While the ATPase activity of BmrA ΔCTh was not significantly reduced compared to the wt (Fig. 2*B*), indicating a dispensable function of this region, the ATPase activity was essentially completely abolished when the entire C-terminus was removed. Next, we aimed to quantify the substrate transport activity of the BmrA variants and used inverted membrane vesicles (IMVs) to determine transport of the hydrophobic dye Hoechst 33342 across the membrane. Similar to the above presented observations, the transport activity of ΔCTh was not significantly reduced compared to BmrA wt, whereas the transport activity of ΔCT was essentially completely abolished (Fig. 2*C*).

Next, the putative effects of C-termini interactions on protein structure and/or conformational changes (IF *versus* OF conformation) were analyzed *via* limited proteolysis. Therefore, IMVs containing the BmrA variants were digested with a low amount of trypsin. In the IF conformation, the transporter is more accessible for trypsin and will be rapidly digested, while in the OF conformation the transporter is more resistant against trypsin digestion (43). In line with this, BmrA wt incorporated in IMVs was digested slower when ATP, Mg^2+^, and orthovanadate were present, which stabilizes the OF conformation (Fig. 2*D*). The proteolytic stability of the truncated variant ΔCT was wt-like, suggesting that the very last C-terminal part is not required for formation and/or stabilization of the OF conformation. In contrast, no increased resistance against trypsin digestion upon the addition of ATP, Mg^2+^ and orthovanadate was observed when the entire C-terminal α-helix was deleted (BmrA ΔCT). In fact, already in absence of ATP, Mg^2+^, and orthovanadate, BmrA ΔCTh was more sensitive against trypsin digestion then the wt (Fig. 2*D*). This indicates that not solely the OF state, yet the overall transporter conformation is destabilized when the full C-terminal BmrA α-helix is deleted. Taken together, the C-terminal residues L569 to L578 are crucial for the BmrA ATPase and transport activity, whereas the amino acids at the very C-terminal end appear to have no direct impact on the ATPase or transport activity of BmrA.

### Cys crosslinking of C-terminal residues indicates structural flexibility

**Figure 3:**
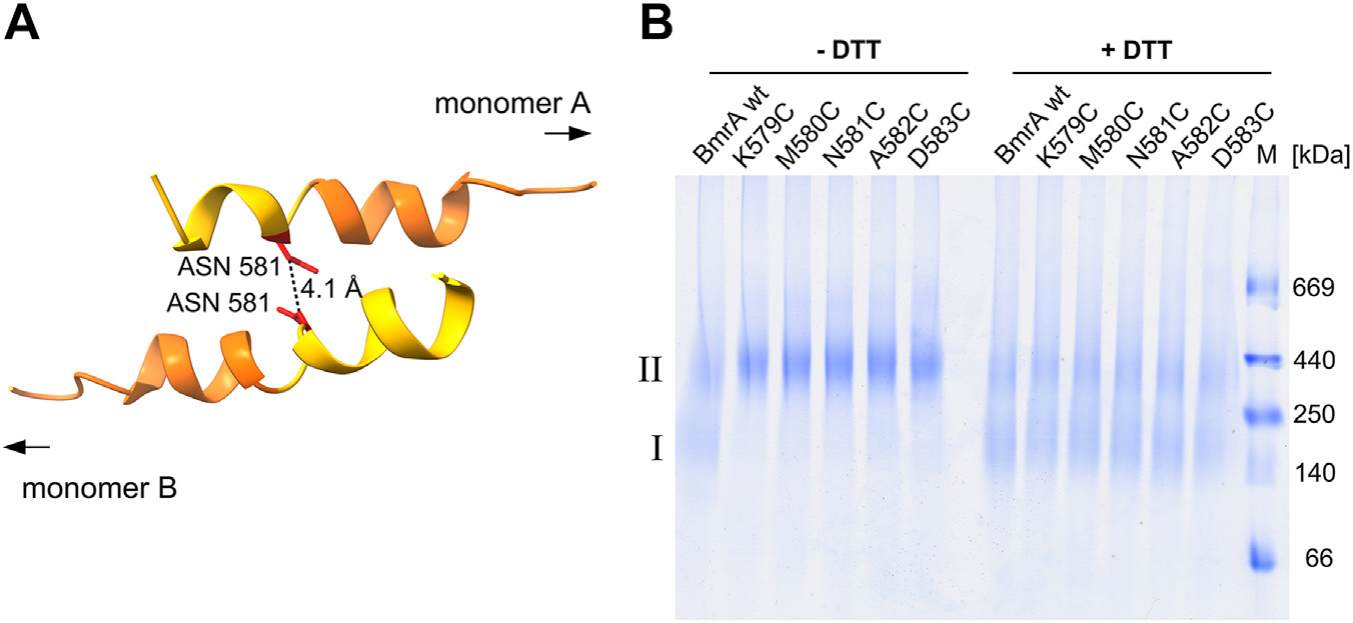
**Antiparallel C-terminal helix dimer of BmrA**. *A*, the C-terminal helices (L569-L578 in *orange*, K579-G589 in *yellow*) of two BmrA monomers (A/B) are in close distance and cross at the amino acid N581 (*red*). The distance of the C_α_ atoms of opposing N581 is about 4.1 Å (pdb: 6R81). *B*, BN-PAGE analysis of BmrA Cys variants cross-linked in DDM micelles. Reducing (+DTT) and oxidizing (−DTT) conditions are indicated. The proteins were separated on a 4 to 16% Bis-Tris gel and monomers (I) and dimers (II) were observed. An SDS PAGE gel with purified proteins is shown in Fig. S1. BmrA, *Bacillus* multidrug resistance ATP; BN, blue native; DDM, n-dodecyl β-D-maltoside.

While the above presented deletion analysis implied that the last part of the C-terminus (K578-G589) has no impact on the BmrA function, in contrast to the first part of the C-terminal α-helix (L569-L578), we studied putative interactions within the dimer, which might be involved in fine-tuning the BmrA activity. The L569-L578 region is located in close proximity to its counterpart from the interacting monomer. Based on the available BmrA structures in the OF conformation, the C-terminal helices of two protomers appear to form an antiparallel helix dimer, and the two helices cross at residue N581 where the distance between the C_α_ residues is only about 4.1 Å (Fig. 3*A*). Noteworthy, in other studies this helix part was found to be not highly structured but more flexible (44). Thus, we aimed at illustrating interaction of the two helix fragments *via* crosslinking the two C-terminal helices of BmrA half-transporters and introduced a Cys residue at position N581. Furthermore, to analyze a potential flexibility in this region, we individually replaced also the residues adjacent to N581 (K579-D583) by Cys and tested whether here formation of disulfide bridges and covalent linkage of two neighboring NBDs is possible also in the absence of ATP. When analyzed *via* nonreducing blue native (BN) PAGE, the wt protein ran as stable monomers and dimers, whereas all Cys-containing variants mainly formed stable dimers in solution (Fig. 3*B*). Yet, in the presence of the reducing agent DTT, also the variants run as monomers and dimers on a BN-PAGE gel, as observed for the wt (Fig. 3*B*). While the C-terminal helices appear to cross at residue N581 in the solved structure, the ability of all here analyzed variants to form cross-linked dimers strongly indicates a certain flexibility of this region as no preferentially interacting residue were identified. Furthermore, the observations indicate that the C-terminal helices of the two transporter halves are in close distance even in absence of ATP, that is, the two NBDs appear to stay in close distance even when no nucleotides are bound.

### Ala scanning of essential C-terminal residues

**Figure 4:**
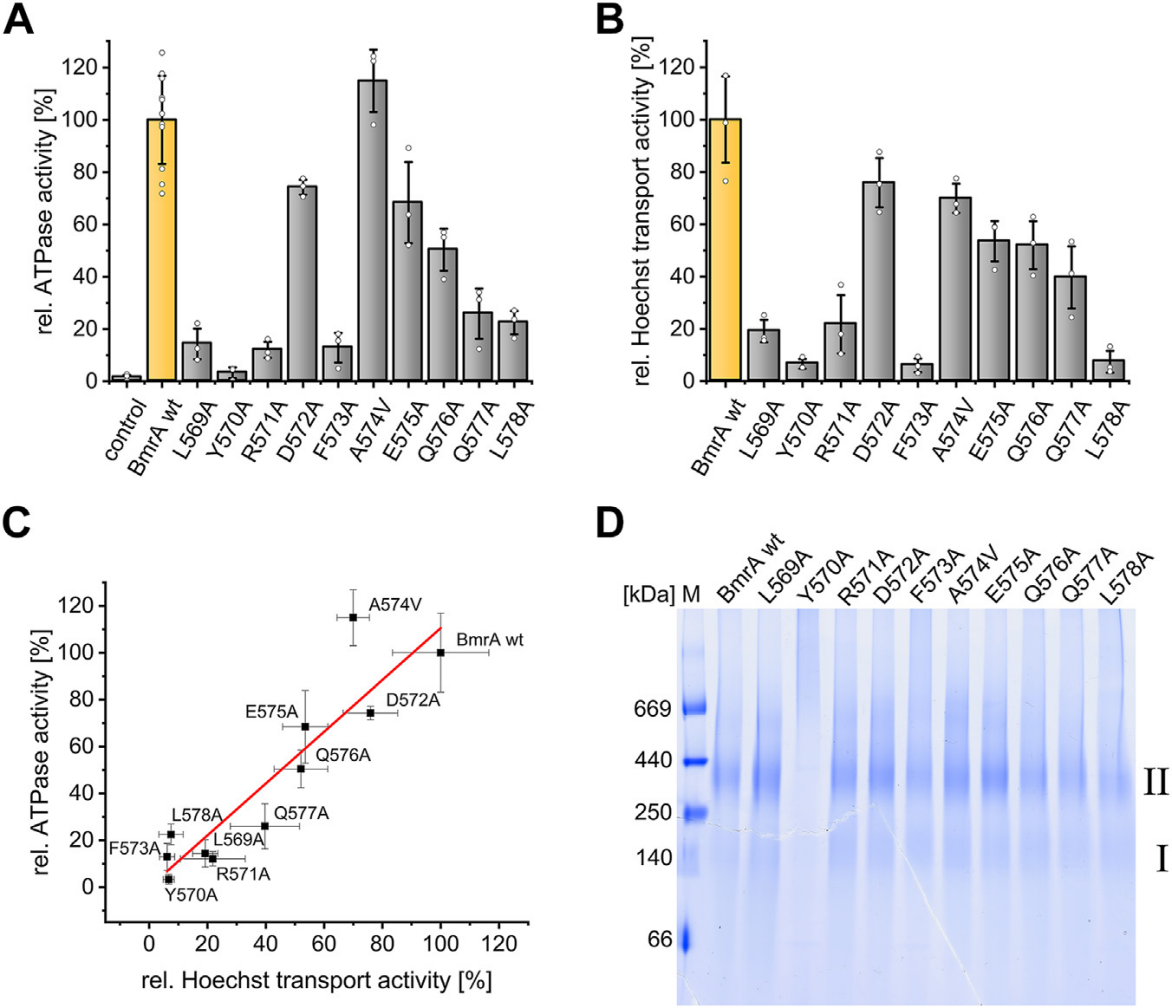
**ATPase, Hoechst 33342 transport activities and dimerization propensities of BmrA wt and variants L569A-L578A**. *A*, mean of relative ATPase activities of the Ala (Val) variants (n = 10 for the wt, n = 3 for the variants, ± SD). Individual data points are shown, representing independent biological replicates. *B*, mean of relative Hoechst 33342 transport activities of the Ala (Val) variants overexpressed in IMVs (n = 3, ± SD). Individual data points are shown, representing independent biological replicates. *C*, relative ATPase activities *versus* the relative transport activities. Data points were fitted with a linear fit shown in *red* (R^2^ = 0.93). *D*, dimerization tendency of BmrA wt and variants in DDM micelles analyzed *via* BN-PAGE. The proteins were separated on a 4 to 16% Bis-Tris gel and the monomer (I) and dimer (II) bands are indicated. BmrA, *Bacillus* multidrug resistance ATP; DDM, n-dodecyl β-D-maltoside; IMV, inverted membrane vesicle.

Based on the results presented above, we next elucidated which amino acids of the essential C-terminal helix fragment (residues L569–L578) are most critical for the ATPase and transport activity of BmrA. For this purpose, all individual amino acids of this region were replaced by Ala (or Val, when an Ala was naturally present), and the ATPase as well as the Hoechst 33342 transport activities of these BmrA variants were tested (Fig. 4). Mutations in the margins of this helical fragment (L569-R571 and Q576-L578) led to strongly reduced (≤25%) ATPase activities compared to the wt activity (Fig. 4*A*).

In contrast, the variants carrying mutations in the helix core (D572A-Q576A) displayed only slightly or moderately reduced ATPase activities (≥50% remaining). The only exception was the variant F573A that showed significantly reduced ATPase activity (as further analyzed and discussed below). Thus, based on these analyses most residues in the core of this helical fragment (residues D572-E575) do most likely not establish contacts in the protein that are crucial for activity, whereas the margin regions L569-R571 and Q576-L578, and potentially F573, are more crucial for the BmrA activity.

Next, we analyzed a putative correlation between the determined ATPase and the relative Hoechst transport activities using IMVs (Fig. 4*B*). The transport activities of five variants (L569A, Y570A, R571A, F573A, and L578A) were less than 25% compared to the BmrA wt activity, whereas the activity of the variants D572A and A574V-Q577A were reduced “only” to about 50% (40% in case of Q577A) of the wt activity. Our results show a very good correlation (Fig. 4*C*) between ATPase and Hoechst transport activities in IMVs considering an uncertainty range of 20%. Since BmrA is functional only as a homodimer, we additionally tested whether the introduced mutations led to an altered dimerization propensity, and analyzed dimerization of the BmrA variants *via* BN-PAGE. As shown in Figure 4*D*, BmrA wt mainly forms dimers in n-dodecyl β-D-maltoside (DDM) micelles, and all here analyzed BmrA variants showed a wt-like dimerization propensity, except Y570A. In contrast to all other variants, BmrA Y570A appears to aggregate, which explains the observed drastically reduced ATPase and transport activities (Fig. 4, *A* and *B*). However, for the remaining variants the reduced activities cannot be explained by an impaired dimerization propensity.

### Thermal stability of the isolated NBD carrying the x-to-Ala mutations

**Figure 5:**
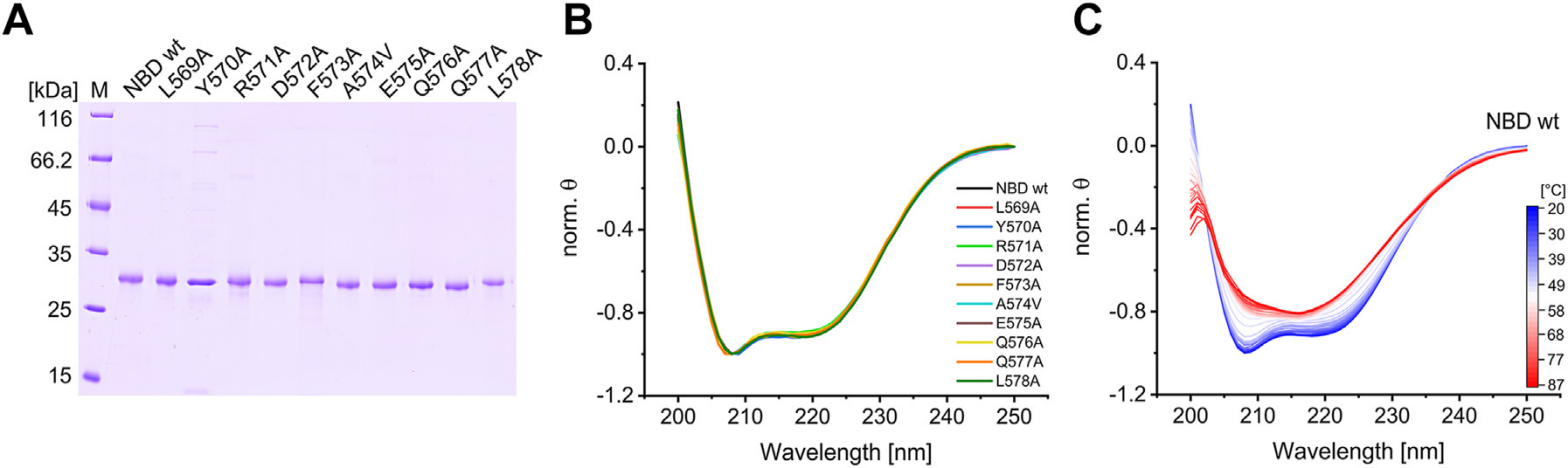
**Secondary structure of NBD wt and the variants with Ala (Val) mutations in the C-terminal helix part L569A-L578A**. *A*, SDS-PAGE analysis of purified NBD variants after Ni-nitrilotriacetic acid (NTA) chromatography and further chromatography steps, if necessary (see methods). The calculated mass for each variant is around 29.5 kDa. M = marker. *B*, CD spectra of NBD wt (*black*), L569A (*red*), Y570A (*blue*), R571A (*light green*), D572A (*violet*), F573A (*ochre*), A574V (*cyan*), E575A (*brown*), Q576A (*yellow*), Q577A (*orange*), and L578A (*dark green*) measured at 25 °C. For better comparison the mean spectra determined for three independent purifications after normalization are shown. The θ value at 250 nm was set to 0 and the minimum θ value to −1. *C*, CD spectra of NBD wt recorded at increasing temperatures (20 °C, *blue* – 88 °C, *red*). For each temperature the mean value determined using three independent purifications was normalized (θ of 250 nm was set to 0; minimum θ was set to −1). BmrA, *Bacillus* multidrug resistance ATP; NBD, nucleotide-binding domain.

While the amino acids were replaced to disturb putative crucial interactions, it was also possible that the replacements affected the overall stability of the NBD. Therefore, we next analyzed the structure and stability of isolated wt and mutated NBDs. For the sake of simplicity, we leave the amino acid numbering of the variants at that of the full-length protein, even if they are NBD variants. *Via* CD spectroscopy, we aimed at identifying potential altered structures of the BmrA variants to exclude that altered folding and/or stability has caused the observed changes in BmrA activities. As shown in Figure 5*A*, all proteins were electrophoretically pure after isolation. The CD spectra of the purified variants measured at 25 °C (Fig. 5*B*) revealed that all variants showed a similar CD spectrum typical for mainly α-helical proteins, with characteristic minima at 208 and 222 nm. Yet, with increasing temperatures (20–88 °C), the protein structure changed, visible as changes in the CD spectra characteristics (Fig. 5*C*, shown for NBD wt as an example and for the other variants in Fig. S2). The CD intensities of both minima increased to a certain degree, and finally solely a minimum at 217 nm was observed at high temperatures, a value typical for β-sheet structures. Using the fluorescence probe thioflavin T (ThT), which changes its fluorescence properties upon binding to amyloid fibrils (45), we observed that its fluorescence emission maximum was significantly increased when the NBD was thermally denatured (Fig. S3). The isolated NBD thus appears to form amyloid-like structures at high temperatures resulting in a high ThT quantum yield at 482 nm.

**Figure 6:**
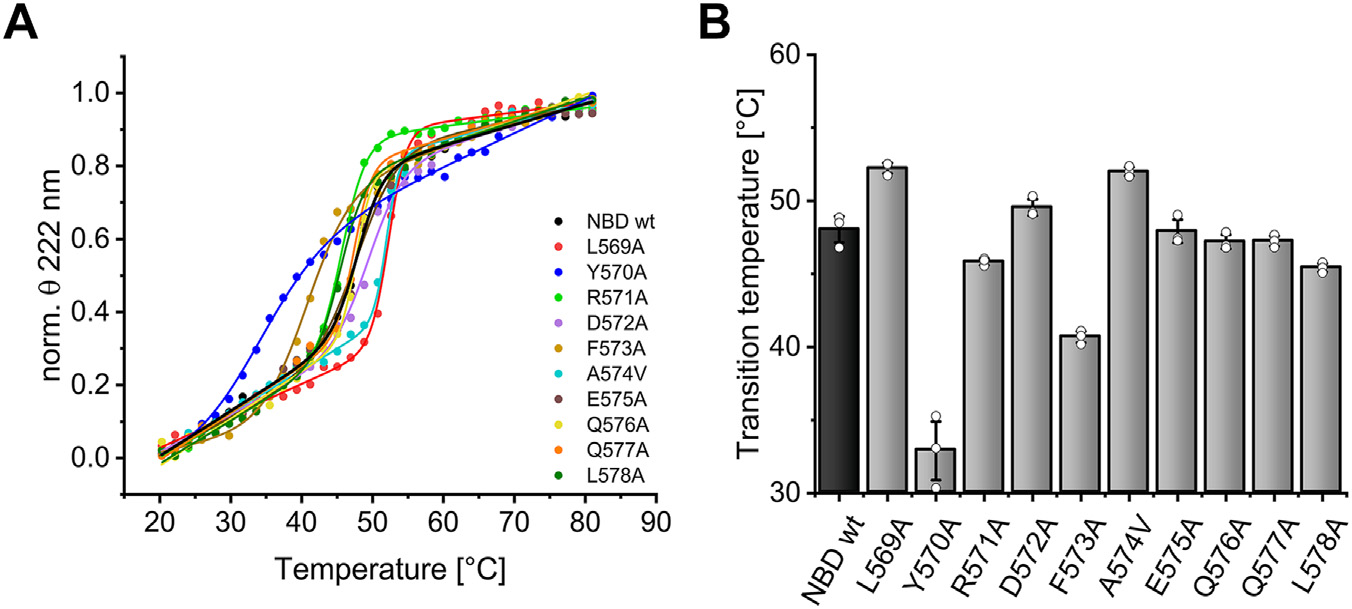
**Transition temperatures of NBD wt and the variant with Ala (Val) mutations in the C-terminal helix part L569A-L578A determined *via* CD measurements**. *A*, normalized θ values at 222 nm of NBD wt (*black*), L569A (*red*), Y570A (*blue*), R571A (*light green*), D572A (*violet*), F573A (*ochre*), A574V (*cyan*), E575A (*brown*), Q576A (*yellow*), Q577A (*orange*), and L578A (*dark green*) plotted against the temperature. The means of three independent measurements of three independent biological replicates are shown, which were fitted with an adapted Boltzmann fit (shown as *lines*, R^2^ > 0.997). *B*, calculated transition temperatures (T_m_) of the NBD variants determined *via* CD spectroscopy. T_m_ corresponds to the inflection points of the fitted curves and is shown with SD: 48.1 ± 0.9 °C (NBD wt), 52.3 ± 0.4 °C (L569A), 32.9 ± 2.0 °C (Y570A), 45.8 ± 0.2 °C (R571A), 49.5 ± 0.6 °C (D572A), 40.7 ± 0.4 °C (F573A), 52.0 ± 0.3 °C (A574V), 47.9 ± 0.8 °C (E575A), 47.7 ± 0.5 °C (Q576A), 47.3 ± 0.4 °C (Q577A), and 45.4 ± 0.3 °C (L578A). NBD, nucleotide-binding domain.

Plotting the ellipticity at 222 nm against the temperature revealed that in most cases denaturation occurred in a highly cooperative manner, with one transition (Fig. 6*A*). In order to estimate the apparent transition temperature, a modified Boltzmann curve was fitted to the data (Fig. 6*A*). The inflection point represents the transition temperature of a protein and can be used to compare the thermal stabilities of proteins and/or protein variants. Most of the NBD variants examined showed comparable transition temperatures, which were in the range of the wt protein ± 4.2 °C (Fig. 6*B*). Yet, for Y570A and F573A the transition temperatures were significantly reduced. For Y570A (T_m_ = 32.9 ± 2.0 °C), the sigmoidal melting curve showed no plateaus at higher and lower temperatures, and thus, we conclude that the structure and stability of this protein is altered due to the amino acid exchange, in line with our observation that the corresponding full-length protein BmrA Y570A tends to aggregate (Fig. 4*D*). Similar observations were made with the variant F573A, where the transition temperature is significantly reduced (40.7 ± 0.4 °C, Fig. 5*B*), explaining the reduced ATPase and transport activity of the respective full-length BmrA variant F573A (Fig. 4, *A* and *B*), although this variant ran as the wt protein during BN-PAGE (Fig. 4*D*). It should be mentioned that all activity measurements were carried out at 25 °C, where no significant structural changes were observed *via* CD measurements (Fig. 5). Taken together, the observations indicate that in case of Y570A and F573A the altered structure/stability of the NBD most likely explains the observed reduced activity of the full-length protein. Yet, F573A still has a wt-like tendency to form dimers at lower temperatures, unlike Y570A (Fig. 4*D*). In contrast, in case of L569A-R571A and L578A, the NBDs had wt-like stability and likely interactions with other parts of the full-length protein are disturbed.

## Discussion

### The C-terminus is crucial for the BmrA ATPase and transport activity

ABC exporter–mediated transmembrane substrate transport involves major conformational changes of the transporter structure, from an IF to an OF conformation and backwards. Here, not only interactions between the TMDs and NBDs are crucial but also interactions between the two NBDs of an ABC transporter to finally form stable dimers resulting in proper ATP hydrolysis and substrate transport. The NBDs of type IV dimeric ABC exporters in the OF conformation are already in quite close proximity (46). Yet, ATP binding is necessary for proper NBD dimerization (47), whereas ATP hydrolysis is needed for the disassembly of the NBD dimer to reset the translocation cycle (48, 49). Based on the recently published BmrA structures in the OF conformation (41) it is apparent that the C-terminal ends are in close proximity and are oriented to some extent side by side (Fig. 1). Consequently, in this study, we investigated a putative role of C-terminal helix interactions for the activity of the *B. subtilis* ABC transporter BmrA.

The C-terminal helix of BmrA can be subdivided into two shorter α-helical fragments (Fig. 1). When the entire helix was deleted, the switch between the IF and OF conformation was disturbed and the ATPase as well as transport activities were completely abolished, indicating a critical role of this helix (Fig. 2). Yet, when solely the very C-terminal helix fragment was deleted (Fig. 2, ΔCTh), the activity was essentially unaltered. Apparently, this helical region does not establish interactions within or between half-transporters crucial for function (Fig. 2). In fact, when we replaced the residues K579-D583 of this fragment individually by Cys, all variants formed stable Cys cross-linked dimers (Fig. 3*B*). Thus, the very C-terminal end of BmrA appears to be highly flexible and is possibly not completely α-helical at all times, in excellent agreement with a recent NMR study of the isolated BmrA NBD monomer (44). Furthermore, in the recently published structures of BmrA in the OF conformation (pdb: 6R81, 6R72, 7OW8, 7BG4), the very C-terminal region appears to be more flexible and has varying α-helix content. This high flexibility indicates that this region does not establish stable and crucial contacts. Our observations clearly support this assumption, as the very C-terminal region is not necessary for the OF state (Fig. 2*D*). Even in the IF conformation, where the NBDs are separated to some degree, the C termini of the BmrA monomers still remain in close proximity, in contrast to other ABC transporter that show a large separation of the NBDs, such as MsbA (42). Close proximity of the NBDs even in the BmrA IF conformation is strongly supported by our results, as our Cys crosslink experiments were carried out in absence of ATP, that is, under conditions favoring the IF conformation.

### A pair of hydrophobic amino acids stabilizes the C-terminal α-helix of BmrA

In contrast to BmrA ΔCTh, the BmrA ΔCT variant showed only a poor ATPase activity, and thus we concluded that some or all amino acids L569-L578 in the C-terminal α-helix are crucial for activity. Based on our mutational analyses, especially the neighboring residues L569-R571 and Q577/L578 are crucial for the overall BmrA function, as well as F573. In contrast, replacement of the remaining core residue of this α-helix fragment (D572, A574-Q576) did not affect the BmrA activity dramatically (Fig. 4). As shown by BN-PAGE analyses, all variants, except Y570A, had a wt-like propensity to dimerize (Fig. 4*D*). This indicates that the Ala substitution substantially altered the stability of the dimer solely in case of Y570A. To elucidate whether the reduced activities of the remaining BmrA variants were due to disturbed putative crucial interactions or a reduced protein stability, we determined the thermal stability of the isolated NBD (variants) *via* CD spectroscopy. Here, we used the soluble NBD since it is properly folded, as shown in numerous studies, and is therefore suitable for our approach (44, 50, 51). Our observations clearly showed that most variants had a wt-like transition temperature, except the NBD variants Y570A and F573A which both showed a drastically reduced transition temperature (Fig. 6). This indicates that both mutations affect the protein stability, possibly due to disruption of an α-helix stabilizing pair of two hydrophobic residues in a distance of three or four amino acids at the N-terminus (52). This nicely explains the observed lowered ATPase and Hoechst 33342 transport activities of the respective full-length variants (Fig. 4). For the other amino acids studied, however, it is more likely that the corresponding effects on protein function are due to disturbed key interactions of the C-terminal helix with crucial structural elements.

### Putative crucial interactions of the C-terminal helix

**Figure 7:**
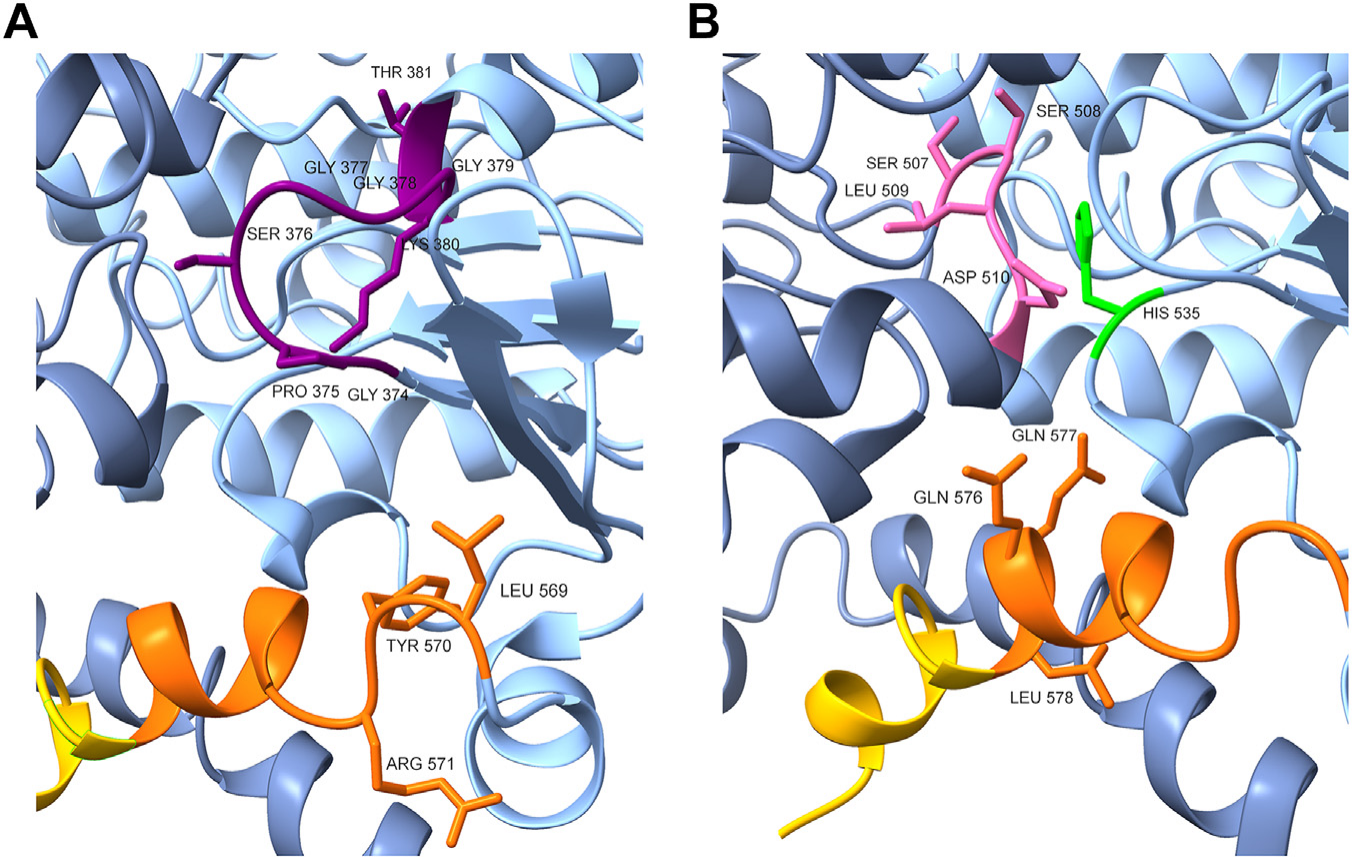
**Structure of BmrA with putative interaction partners of crucial residues at the C-terminal end**. View on the C-terminal α-helix of a BmrA dimer, where one monomer is colored in *blue* and the second in *dark blue*. Of one monomer, the first segment of the C-terminal α-helix (L569-L578) is highlighted in *orange* and the second segment (K578-G589) in *yellow* with certain amino acids marked. Additionally, the amino acids residues of (*A*) the Walker A motif (*purple*), or (*B*) of the H-loop (*green*) and the D-loop (*pink*) are shown (pdb: 6R81). BmrA, *Bacillus* multidrug resistance ATP.

Based on the structures of the BmrA full-length protein (pdb: 6R81, 6R72, 7OW8, 7BG4, 8QOE), the residues L569-R571 are in close proximity to the Walker A motif of the same monomer (Fig. 7*A*). The Walker A motif mediates ATP binding and triggers NBD dimerization (23). Therefore, it is feasible to assume that mutating the residues L569-R571 individually to Ala affects dimerization and ATP hydrolysis of BmrA. A decreased ATPase activity could also be determined for the variant carrying mutations in Q576-L578 (Fig. 4). These amino acids are in close distance (C_α_<10 Å) to residues of the H-loop of the same monomer (Fig. 7*B*). This loop recognizes and adjusts ATP and is, consequently, also needed for proper dimerization and ATP hydrolysis by an ABC transporter. For example, mutations in the eponymous H-loop caused a drastically decreased ATPase activity of the ABC transporter HisP, MalK, and HlyB, and substrate transport activities were not observed anymore (35, 53, 54). The lack of crucial interaction partners for amino acid residues that directly interact with the conserved (eponymous) His of the H-loop could lead to further malfunctions of this important structural part. Furthermore, the amino acid residues Q576 and Q577 are close to the D-helix of the adjacent NBD (Fig. 7*B*). In line with this, the C-terminal α-helix is crucially involved in dimerization of the eukaryotic ABC transporter TAP1 (55). Thus, interactions between the C-terminal amino acid residues Q576 to L578 and the H-loop of the same monomer as well as the D-helix of the opposite NBD are potentially important for dimerization of BmrA, and thereby for ATP hydrolysis.

In summary, the N-terminal part of the last C-terminal α-helix of BmrA is important for the ABC transporter to be fully functional, likely due to interactions with conserved elements crucial for ATP binding and/or hydrolysis. Especially the proximity of the residues L569-R571 to the Walker A motif and the interactions of Q576-L578 with residues interacting with the H-loop of the same NBD and the D-helix of the adjacent NBD might be crucial for mediating NBD dimerization and thereby regulating the ATPase activity of BmrA.

## Experimental procedures

### Cloning

For heterologous expression of full-length BmrA or the isolated BmrA NBD with an C-terminal His_6_-tag in *Escherichia coli*, the *BmrA* encoding gene of *B. subtilis* strain 168 was amplified *via* PCR using genomic DNA as a template and subsequently cloned into the expression plasmid pET303-CT/His (Invitrogen), as described in detail recently (56). Individual or clusters of amino acids were mutated *via* site-directed mutagenesis using the oligonucleotides listed in Table 1.

**Table 1:** Oligonucleotides used in this study

Primer	5′-sequence-3′
BmrA-linker XhoI K579 rev	GCTACCCTCGAG**ACCACCAGAACCACC**CAGCTGCTGTTCAGCAAAATC
BmrA-linker XhoI L569 rev	GCTACCCTCGAG**ACCACCAGAACCACC**GCCATGGGAAGCCATTAAC
QC BmrA L569A fw	GTTAATGGCTTCCCATGGC**GCG**TACCGGGATTTTGCTG
QC BmrA L569A rev	CAGCAAAATCCCGGTA**CGC**GCCATGGGAAGCCATTAAC
QC BmrA Y570A fw	GCTTCCCATGGCCTT**GCG**CGGGATTTTGCTGAAC
QC BmrA Y570A rev	GTTCAGCAAAATCCCG**CGC**AAGGCCATGGGAAGC
QC BmrA R571A fw	GCTTCCCATGGCCTTTAC**GC**GGATTTTGCTGAACAG
QC BmrA R571A rev	CTGTTCAGCAAAATCC**GC**GTAAAGGCCATGGGAAGC
QC BmrA D572A fw	CATGGCCTTTACCGGG**CG**TTTGCTGAACAGCAG
QC BmrA D572A rev	CTGCTGTTCAGCAAA**CG**CCCGGTAAAGGCCATG
QC BmrA F573A fw	GGCCTTTACCGGGAT**GCG**GCTGAACAGCAGCTG
QC BmrA F573A rev	CAGCTGCTGTTCAGC**CGC**ATCCCGGTAAAGGCC
QC BmrA A574V fw	CTTTACCGGGATTTTG**TG**GAACAGCAGCTGAAAATG
QC BmrA A574V rev	CATTTTCAGCTGCTGTTC**CA**CAAAATCCCGGTAAAG
QC BmrA E575A fw	CTTTACCGGGATTTTGCTG**CG**CAGCAGCTGAAAATG
QC BmrA E575A rev	CATTTTCAGCTGCTG**CG**CAGCAAAATCCCGGTAAAG
QC BmrA Q576A fw	CGGGATTTTGCTGAA**GC**GCAGCTGAAAATGAATGCG
QC BmrA Q576A rev	CGCATTCATTTTCAGCTGC**GC**TTCAGCAAAATCCCG
QC BmrA Q577A fw	GATTTTGCTGAACAG**GC**GCTGAAAATGAATGCG
QC BmrA Q577A rev	CGCATTCATTTTCAGC**GC**CTGTTCAGCAAAATC
QC BmrA L578A fw	GATTTTGCTGAACAGCAG**GC**GAAAATGAATGCGGAC
QC BmrA L578A rev	GTCCGCATTCATTTTC**GC**CTGCTGTTCAGCAAAATC
QC BmrA K579C fw	GCTGAACAGCAGCTG**TGC**ATGAATGCGGACTTAG
QC BmrA K579C rev	CTAAGTCCGCATTCAT**GCA**CAGCTGCTGTTCAGC
QC BmrA M580C fw	GAACAGCAGCTGAAA**TGC**AATGCGGACTTAGAAAAC
QC BmrA M580C rev	GTTTTCTAAGTCCGCATT**GCA**TTTCAGCTGCTGTTC
QC BmrA N581C fw	CAGCAGCTGAAAATG**TGC**GCGGACTTAGAAAAC
QC BmrA N581C rev	GTTTTCTAAGTCCGC**GCA**CATTTTCAGCTGCTG
QC BmrA A582C fw	CAGCTGAAAATGAAT**TGC**GACTTAGAAAACAAAGCC
QC BmrA A582C rev	GGCTTTGTTTTCTAAGTC**GCA**ATTCATTTTCAGCTG
QC BmrA D583 C fw	CTGAAAATGAATGCG**TG**CTTAGAAAACAAAGCC
QC BmrA D583C rev	GGCTTTGTTTTCTAAG**CA**CGCATTCATTTTCAG
BmrA NBD *Xba*I fw	GCGCGCTCTAGAATGAAACAAATTGAAAATGCAC
BmrA NBD *Xho*I re	GGCCATCTCGAGCCCGGCTTTGTTTTCTAAGTCC

BmrA, *Bacillus* multidrug resistance ATP; NBD, nucleotide-binding domain.

Highlighted in bold are the mutated bases. Sequences recognized by a restriction enzyme used for cloning are underlined.

### Expression and purification

After heterologous expression in *E. coli*, full-length BmrA wt and variants were purified in presence of detergent (DDM), and the soluble NBD variants without detergent, as described in detail in (51, 56). Due to a low expression level and insufficient purification *via* solely a metal chelate affinity column, the isolated NBDs carrying the mutations R241A, F243A, Q246A, Q247A, or L248A were further purified *via* size-exclusion chromatography using a Superose 12 10/300 GL column (Cytiva). The column was equilibrated with 50 mM NaH_2_PO_4_/Na_2_HPO_4_, 150 mM NaCl, 10% glycerol, pH = 8.0. Main peak fractions were then collected and concentrated. The NBD Y240A protein was further purified *via* anion-exchange chromatography using a self-packed Q Sepharose Fast Flow column (Cytiva). Phosphate buffer was used for column equilibration and the protein was eluted at a concentration of 100 mM NaCl.

The protein concentration was determined *via* absorption spectroscopy. For the BmrA wt, NBD wt and most analyzed variants, the extinction coefficient at 280 nm was either ε_BmrA wt_ = 38,850 M^−1^ cm^−1^ or ε_NBD wt_ = 15,930 M^−1^ cm^−1^, computed using ExPASy’s ProtParam tool (57). For some variants different coefficients were calculated: ε_BmrA ΔCT; BmrA Y570A_ = 37,360 M^−1^ cm^−1^, ε_NBD ΔCT, NBD Y570A_ = 14,440 M^−1^ cm^−1^, and ε_BmrA K579C-D583C_ = 38,975 M^−1^ cm^−1^.

### BN PAGE analysis

Oligomerization of BmrA variants in detergent was analyzed *via* BN-PAGE. Proteins samples were prepared in BN-PAGE sample buffer (50 mM Bis-Tris, 50 mM NaCl, 10% (w/v) glycerol, 0.001% Ponceau S, pH = 7.2). In case of Cys-containing BmrA variants, 0.25 mg/ml of purified proteins were preincubated ± 100 μM DTT for 30 min at room temperature (RT). Afterward, DDM was added to the reduced or non-reduced proteins to reach a final DDM concentration of 1%. Immediately before electrophoresis, Coomassie brilliant blue G-250 (final conc. 1%) was added to the samples. Four micrograms protein per lane were separated on 4 to 16% Bis-Tris polyacrylamide gels (Invitrogen) using the cathode buffer (50 mM Bis-Tris, 50 mM tricine, 0.02% Coomassie brilliant blue G-250) and the anode buffer (50 mM Bis-Tris, 50 mM tricine, pH = 6.8). Electrophoresis was performed at first for 30 min at 150 V, followed by 90 min at 250 V. Separated proteins were fixed in 40% methanol and 10% acetic acid and stained with Coomassie brilliant blue G-250. BN-PAGE gels were scanned with the gel scanner ViewPix 700 (Biostep GmbH). The Amersham High Molecular Weight Calibration Kit for native electrophoresis (GE Healthcare) was used for molecular weight estimation.

### ATPase activity assay

The *in vitro* ATPase activity of the purified proteins was determined in DDM micelles using a coupled and regenerative assay, as recently described (56). The ATPase activity of BmrA wt and the different variants (0.2 μM) was determined at 25 °C in 50 mM Hepes-KOH (pH 8.0) with 3.5 mM Na_2_ATP, 10 mM MgCl_2_, 0.28 mM NADH, and 2 mM phosphoenolpyruvate. The ATPase activity of the variants is expressed relative to BmrA wt (=100%), and the corresponding error bars were calculated based on error propagation.

### Limited proteolysis of BmrA variants

BmrA wt and variants were overexpressed in *E. coli* C41(DE3) cells, which were used to prepare IMVs as described in (56). 2 μg/μl IMVs were preincubated in 20 mM Tris–HCl, 1 mM EDTA, pH = 8.0 in presence or absence of 2 mM ATP, 1 mM orthovanadate, and 3 mM MgCl_2_ for 15 min at room temperature. Prior to proteolysis, a sample for SDS-PAGE analysis was secured, and sample buffer was added (final conc. 50 mM Tris–HCl (pH 6.8), 10% (v/v) glycerol, 2% SDS (w/v), and 0.04% (w/v) bromophenol blue). Proteolysis was started by adding 1 μg trypsin per 250 μg IMVs (protein content), samples were collected for an SDS-PAGE analysis after 2, 5, 15, 30, 60, 120, and 180 min, and SDS-PAGE sample buffer was added. To inactivate trypsin, the SDS PAGE samples were immediately incubated for 10 min at 100 °C and stored afterward at −20 °C until electrophoresis. IMVs (16 μg/lanes) were separated on a 10% SDS-PAGE gel and stained after electrophoresis with Coomassie brilliant blue R520. The Pierce Unstained Protein MW Marker (Thermo Fisher Scientific) was used as a protein standard.

### Hoechst 33342 transport activity assay

IMVs were prepared and the transport activity of BmrA variants was measured as described in detail in (56). By comparing the band intensity of the overexpressed variants in the SDS-PAGE with the one of BmrA wt (using ImageJ; https://imagej.net/ij/ (58)), the relative expression level was determined. The measured transport activities were corrected accordingly, assuming a linear correlation between activity and concentration. The transport activity of the variants is expressed relative to BmrA wt (=100%), and the corresponding error bars were calculated based on error propagation.

### CD spectroscopy

A JASCO J-1500 CD spectrometer and the MPTC-490S temperature-controlled cell holder (JASCO cooperation) were used for recording CD spectra of 0.4 mg/ml protein in 50 mM NaH_2_PO_4_/Na_2_HPO_4_, 150 mM NaCl, and 10% glycerol, pH 8.0. Instrument settings were: scan rate 100 nm/min, 1 mm cell length, 1 nm steps, 1 nm bandwidth, 1 s data integration time, and 6-time accumulation at 25 °C. In case of measuring the thermal stability between 20 to 88 °C (2 °C steps with a heating rate of 1 °C/min), only one spectrum was measured with the same settings. The spectra were smoothed using the Savitzky–Golay filter implemented in the instrument's software. For each protein three samples from three independent purifications were measured, averaged and normalized to yield the final denaturation curve. Using the program Origin (version 9.60) (OriginLab Corporation), the data points were fitted with an adapted Boltzmann fit (Equation 1), which allowed linear slopes in the plateau areas of the curve, and the transition temperature (T_m_) was determined.

(1) $$\theta_{222\;nm}\left(T\right)=\frac{\left(T*\;m_{N}+\theta_{N}\right)-\left(T*\;m_{D}+\theta_{D}\right)}{1+e^{\frac{T-T_{m}}{dT}}}+\left(T*m_{D}+\theta_{D}\right)$$

*θ*_222 nm_ = measured ellipticity at 222 nm; *T* = temperature; *θ*_*N*_/*θ*_*D*_ = ellipticities of native/denatured protein at plateau regions; *m*_*N*_/*m*_*D*_ = slopes of plateaus.

### ThT binding to amyloid-like structures formed by the NBD

ThT was purchased from Merck KGaA and dissolved in water. The solution was filtered through a 0.2 μM filter and afterwards the concentration was determined by measuring the absorbance at 416 nm (ε = 22,620 M^-1^ cm^-1^). Stock solutions of 50 μM were prepared and stored at −20 °C. The isolated NBD wt was heated up to 88 °C (1 °C/min). Preheated and nontreated NBD was then incubated at a concentration of 0.4 mg/ml with 1 μM ThT for 1 h at room temperature. Emission spectra were recorded between 465 to 565 nm (slit widths 5 nm) upon excitation at 450 nm (slit widths 5 nm) at 20 °C using the FluoroMax-4 fluorometer (Horiba Instruments Inc).

## Data availability

All discussed data are presented within the article.

## Supporting information

This article contains supporting information.

## Conflict of interest

The authors declare that they have no conflicts of interest with the contents of this article.
