# Supplementary material for: The C-terminal α-helix is crucial for the activity of the bacterial ABC transporter BmrA
Source: J Biol Chem. 2024 Dec 18;301(2):108098. doi: 10.1016/j.jbc.2024.108098 (PMC11774805; doi:10.1016/j.jbc.2024.108098)
Supplement: Supplemental data [file mmc1.pdf]

Supporting Information

The C-terminal α-helix is crucial for the activity of the bacterial ABC transporter BmrA

Veronika Osten[1,#], Kristin Oepen[1,#] and Dirk Schneider[1,2,*]

[1]Department of Chemistry – Biochemistry and [2]Institute of Molecular Physiology, Johannes Gutenberg-University, 55099 Mainz, Germany

[#]These authors contributed equally

*To whom correspondence should be addressed:

Dirk Schneider, Department of Chemistry - Biochemistry, Johannes Gutenberg-University, Hanns-Dieter-Hüsch-Weg 17, 55128 Mainz, Germany

phone: +49 6131 39-55833, fax: +49 6131 39-25348; e-mail: Dirk.Schneider@uni-mainz.de

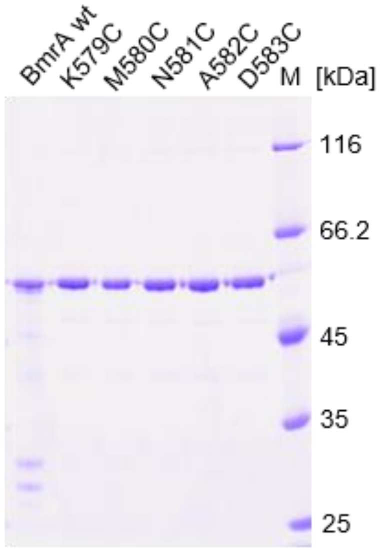

Figure S1: Purified BmrA wt and Cys-variants analyzed.

SDS-PAGE analysis of purified BmrA Cvs-variants. The calculated mass for each variant is around 65.6 kDa. M = marker.

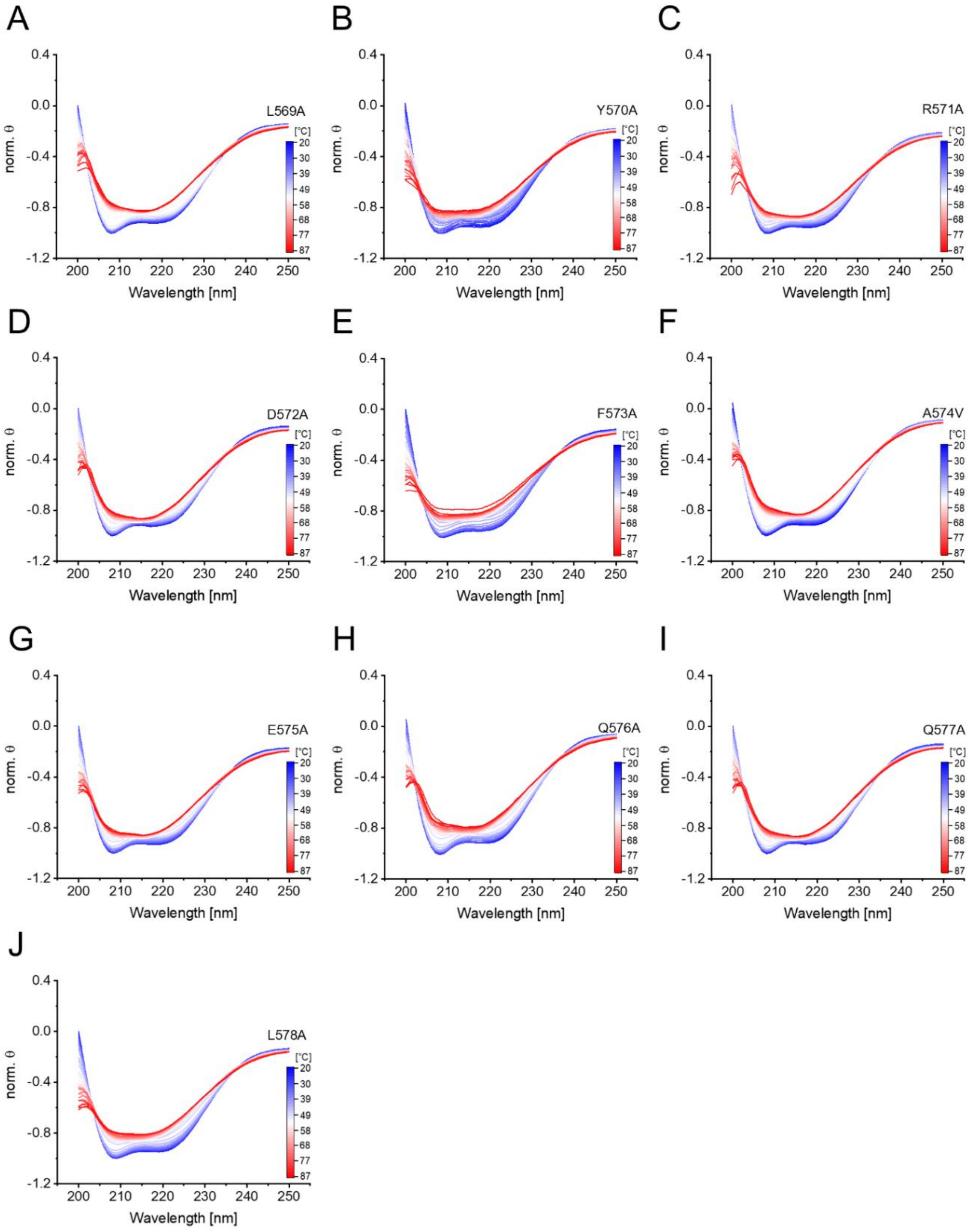

Figure S2: Thermal denaturation of the NBD variants L569A-L578A.

CD spectra of the NBD variants (A) L569A, (B) Y570A, (C) R571A, (D) D572A, (E) F573A, (F) E575A, (H) Q576A, (I) Q577A and (J) L578A recorded at increasing temperatures (20 °C, blue – 88 °C, red). For each temperature the mean value determined using three independent purifications was normalized (θ of 250 nm was set to 0; minimum θ was set to -1).

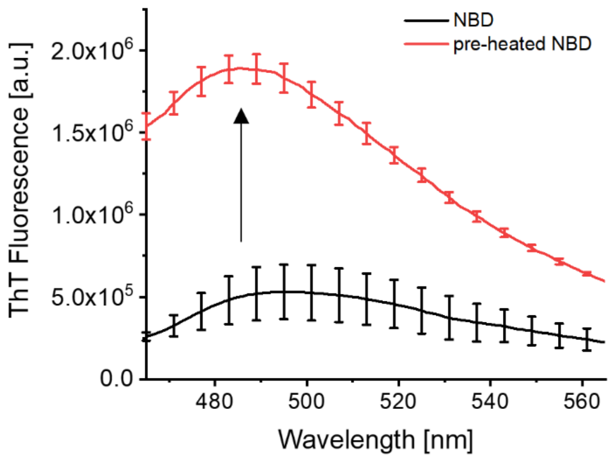

Figure S3: ThT binding to (pre-heated) NBD suggests the formation of amyloid-like structures.

Emission spectra of Thioflavin T after incubation with untreated (black) or pre-heated NBD (red). The fluorescence emission between 465-565 nm (slit width 5 nm) was recorded upon excitation at 450 nm (slit width 5 nm). Shown are the mean spectra of measurements of three independent purification with SD and the increase of Thioflavin T fluorescence at 482 nm is indicated.
